# Long-term warming results in species-specific shifts in seed mass in alpine communities

**DOI:** 10.7717/peerj.7416

**Published:** 2019-07-31

**Authors:** Chunhui Zhang, Zhen Ma, Huakun Zhou, Xinquan Zhao

**Affiliations:** 1 State Key Laboratory of Plateau Ecology and Agriculture, Qinghai University, Xining, Qinghai, China; 2 Key Laboratory of Restoration Ecology for Cold Regions in Qinghai Province, Northwest Institute of Plateau Biology, Chinese Academy of Sciences, Xining, Qinghai, China; 3 Key Laboratory of Adaptation and Evolution of Plateau Biota, Northwest Institute of Plateau Biology, Chinese Academy of Sciences, Xining, Qinghai, China

**Keywords:** Climate change, Functional traits, Grazing, Qinghai-tibet plateau, Trait variation

## Abstract

**Background:**

Global warming can cause variation in plant functional traits due to phenotypic plasticity or rapid microevolutionary change. Seed mass represents a fundamental axis of trait variation in plants, from an individual to a community scale. Here, we hypothesize that long-term warming can shift the mean seed mass of species.

**Methods:**

We tested our hypothesis in plots that had been warmed over 18 years in alpine meadow communities with a history of light grazing (LG) and heavy grazing (HG) on the Qinghai-Tibet plateau. In this study, seeds were collected during the growing season of 2015.

**Results:**

We found that warming increased the mean seed mass of 4 (*n* = 19) species in the LG meadow and 6 (*n* = 20) species in the HG meadow, while decreasing the mean seed mass of 6 species in the LG and HG meadows, respectively. For 7 species, grazing history modified the effect of warming on seed mass. Therefore, we concluded that long-term warming can shift the mean seed mass at the species level. However, the direction of this variation is species-specific. Our study suggests that mean seed mass of alpine plant species appears to decrease in warmer (less stressful) habitats based on life-history theory, but it also suggests there may be an underlying trade-off in which mean seed mass may increase due to greater thermal energy inputs into seed development. Furthermore, the physical and biotic environment modulating this trade-off result in complex patterns of variation in mean seed mass of alpine plant species facing global warming.

## Introduction

Global warming induces variation in plant functional traits (Nicotra et al., 2010; Anderegg, 2015; Bjorkman et al., 2018). For example, experimental warming resulted in taller plants and larger leaves in Arctic tundra (Hudson, Henry & Cornwell, 2011; Bjorkman et al., 2018). Such temperature-driven changes in plant traits may reflect either phenotypic variation or rapid microevolutionary change (Thompson, 2013; Merilä & Hendry, 2014).

Seed mass influences temporal and spatial seed dispersal, seedling survival, and the number of seeds that can be produced for a given amount of energy (Fenner & Thompson, 2005; Moles et al., 2005; Moles et al., 2007). Therefore, it represents a fundamental axis of trait variation in plants, from an individual to a community scale (Westoby et al., 2002; Muller-Landau, 2003; Wright et al., 2007). Theoretically, warming may induce variation in the seed mass of species, although seed mass is generally considered to exhibit relative intraspecific constancy (Harper, Lovell & Moore, 1970; Marshall, Levin & Fowler, 1986; Michaels et al., 1988; Fenner, 1992; Albert et al., 2012).

If air temperature increases, warming may provide greater energy for seed development (Ozkan, Akcaoz & Fert, 2004), and prolonged growing seasons due to warming may lengthen the period of seed development (Sherry et al., 2007), especially in alpine environments where growing seasons are short and air temperatures are low (Körner, 2003; Klein, Harte & Zhao, 2004). Previous studies found that shot-term (1–3 year) experimental warming have a positive effect on mean seed mass (i.e., weight per seed) of *Dryas octopetala* (Wookey et al., 1995), *Ranunculus acris* (Totland, 1999), *Eriophorum vaginatum* (Molau & Shaver, 1997), *Saxifraga stellaris* (Sandvik, 2001), *Ranunculus glacialis* (Totland & Alatalo, 2002), *Parnassia palustris* (Sandvik & Eide, 2009), *Anemone nemorosa* (De Frenne et al., 2011), *Cardamine hirsuta* (Cao et al., 2016), *Koenigia islandica* (Cui et al., 2017). A recent meta-analysis also found a strong thermal memory for seed mass using thirty species including *Arabidopsis*, wild and domesticated species from thirty references (Fernández-Pascual, Efisio & Pritchard, 2019). Therefore, we predict that seeds of species are predicted to become larger in warming environments in this study (termed hypothesis I).

Alternatively, life-history theory proposes a trade-off between seed number and size (i.e., Smith-Fretwell model), i.e., allocation of a given quantity of resource into fewer, larger seeds versus into many, smaller ones (Smith & Fretwell, 1974; Fenner & Thompson, 2005). Larger-seeded species are considered to be superior competitors and stress-tolerators during establishment (Muller-Landau, 2003; Fenner & Thompson, 2005). Life-history theory predicts that the minimal maternal investment requirement (i.e., seed mass) may be higher/lower, when the environment becomes harsher/superior (Leishman et al., 2000; Zhang, 2004; Fenner & Thompson, 2005; Silvertown & Charlesworth, 2009). For example, low soil fertility significantly increased mean seed mass of *Vigna unguiculata* from 133 mg to 165 mg (Kahn & Stoffella, 1985). Equally, the mean seed mass (0.55 mg) of *Ranunculus reptans* in areas of high density tended to be higher than its mean seed mass (0.51 mg) in areas of low density (Van Kleunen, Fischer & Schmid, 2001). Consequently, we predict that the minimal maternal investment requirement becomes lower when the environment warms (i.e., in a less stressful environment) in alpine environments of the Qinghai-Tibet Plateau, where plants growth and seeds production are strongly limited by low temperatures and facilitation is the dominant interaction within alpine communities (Körner, 2003; Chu et al., 2008; Wang et al., 2008). In other words, we predict that mean seed mass of species decreases in warming environments in this study (termed hypothesis II).

If both mechanisms behind hypothesis I and II exist, their combined effects on seed mass may result in mixed results (i.e., increase, no change or decrease in mean seed mass). Hovenden et al. (2008) found that mean seed mass was not significantly affected by 4-year warming in any of 15 species in temperate grasslands of southeastern Tasmania, Australia. However, there is no empirical study to support hypothesis II.

Grazing, as the main land use and disturbances in alpine communities, has profound influences on physical environment and biotic interactions. Thus, grazing may modify the effect of warming on seed mass. Here, we tested our hypotheses in 18-year warming plots in alpine communities with a light grazing history and heavy grazing history.

## Materials & Methods

### Study region

The study was performed at the Haibei Research Station (37°37′N, 101°12′E; elevation: 3,200 m) in the northeastern Qinghai-Tibet plateau. Mean annual temperature is −1.7 °C, ranging from −15.2 °C in January to 9.9 °C in July (Klein, Harte & Zhao, 2004). Mean annual, largely summer, precipitation is 561 mm (Klein, Harte & Zhao, 2004). The growing season generally ranges from May to September (Klein, Harte & Zhao, 2004). Soil type is Mollic-Cryic Cambisols (Klein, Harte & Zhao, 2004). The grassland types are mainly alpine meadow. The Qinghai-Tibet plateau also has a history of seasonal grazing dating back thousands of years (Zheng, Zhang & Wu, 2000). In recent decades, heavy grazing has caused large-scale rangeland degradation (Ma et al., 2014). We identified two sites (about 1.5 km apart) with “low” and “high” grazing intensity histories respectively before starting the experiment, namely the low grazing intensity history meadow site (LG meadow) and the high grazing intensity history meadow site (HG meadow). We assigned these qualitative history labels after interviewing local herders and senior researchers about land use patterns and research history at these sites beginning in 1982 (Klein, Harte & Zhao, 2004). The “high grazing history site” had both more animals (i.e., sheep) per unite area and animals grazing for a longer duration of time (Klein, Harte & Zhao, 2004). Therefore, both the grazing intensity and grazing duration differed among the grazing history sites. The low and high grazing history sites were similar in other features —such as slope, aspect and soil type. Further environmental details about the study region can be found in Klein, Harte & Zhao (2004) and Zhang et al. (2017).

### Experimental design and data collection

**Figure 1:**
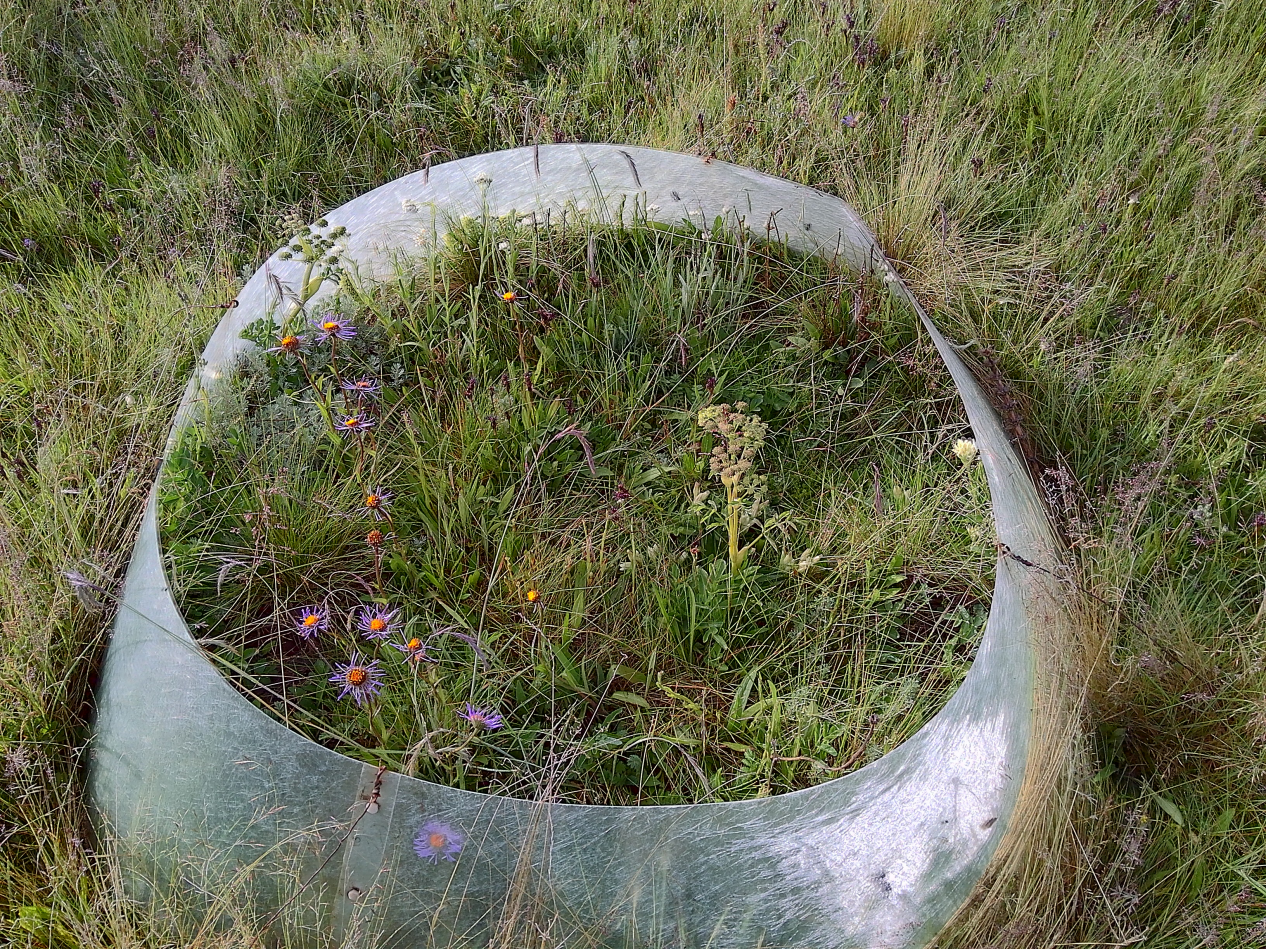
A photograph of a fiberglass open top chamber (OTC) in the field.

In 1997, the two field sites measuring 30 m ×30 m were located on a flat slope (<1°) and were fenced off from grazers. Warming was simulated using fiberglass open top chambers (OTCs) in two field sites from 1997 (see schematic representation of the experiment design in Fig. S1). The OTCs, which were 1.5 and 0.75 m in diameter at the base, and the top, respectively, and 0.40 m high (see Fig. 1), are used by the International Tundra Experiment (Molau & Mølgaard, 1996) and are commonly employed to study the effects of climate warming on plant traits, biotic interactions, community structure and functions (Arft et al., 1999; Walker et al., 2006; Liu et al., 2011; Elmendorf et al., 2012; Sistla et al., 2013; Ylänne, Stark & Tolvanen, 2015; Liu et al., 2016; Rousk, Michelsen & Rousk, 2016). Each site had eight plots of both warm and control treatments. The OTCs remained on the plots year-round. During the growing season, the OTCs increased the average daily air temperature by 1.0–2.0 °C (Klein, Harte & Zhao, 2005).

During the growing season of 2015, seeds were collected from five ramets for the studied species in warmed and control treatments, respectively. Seeds were air-dried to a constant mass at room temperature (approximately 15 °C) before being weighed (Manning, Houston & Evans, 2009; Pivatto et al., 2014; Qi et al., 2014; Zhang et al., 2014a; Zhang et al., 2014b). Seed mass was defined as the weight of the embryo, endosperm and seed coat or fruit coat (e.g., Asteraceae seeds of which we cannot separate fruit coat from seed coat). Accessories (e.g., wings, comas, pappus, elaiosomes, fruit flesh) were not included in measures of seed mass (Cornelissen et al., 2003). Total seed mass was weighed and seed number was counted for each of five individuals of each species, and then mean seed mass was computed for each individual of each species. Our seed mass dataset includes 19 species in both warmed and control plots in the LG site and 20 species in both warmed and control plots in the HG site (see Tables S1–S3 for their seed mass, seed number and associated characteristics, separately).

See details of the experimental design and microclimate effects of the OTCs in Klein, Harte & Zhao (2004) and Klein, Harte & Zhao (2005) and Zhang et al. (2017).

Soil samples were collected at the end of August 2015. Three two cm-diameter samples from each of the six plots for control treatment at two site were divided into three layers of 0–10 cm and 10–20 cm, and soil from the same depth from a plot was pooled. All soil samples (2 depths ×6 plots ×2 sites) were analyzed for total nitrogen, available nitrogen, total phosphorus, available phosphorus and organic carbon content. These soil characteristics were analyzed using the methods described by Zhang et al. (2019).

### Statistical analyses

Prior to analyses, mean seed mass (mg) was ln-transformed to optimize normality of frequency distributions.

A linear mixed-effect model was fitted using the R-package nlme (Pinheiro et al., 2018) with warming treatment and species and their interaction as the fixed effects and plot (nested within site) and site as the random effects to assess the effect of warming on seed mass across all species in both sites. We used Wilcoxon rank sum tests to assess the effect of warming on mean seed mass for each of all species in each site, separately. To examine the main and interactive effects of warming treatment and site on mean seed mass for the species that were found in both LG and HG sites, we conducted a single two-way analysis of variance (ANOVA). Significant interactions meant that the effect of warming on mean seed mass differed between sites. T-tests were used to test if there are significant differences of soil properties of 0–10 cm and 10–20 cm in LG and HG meadow sites. All analyses were conducted in R (http://www.R-project.org).

## Results

**Figure 2:**
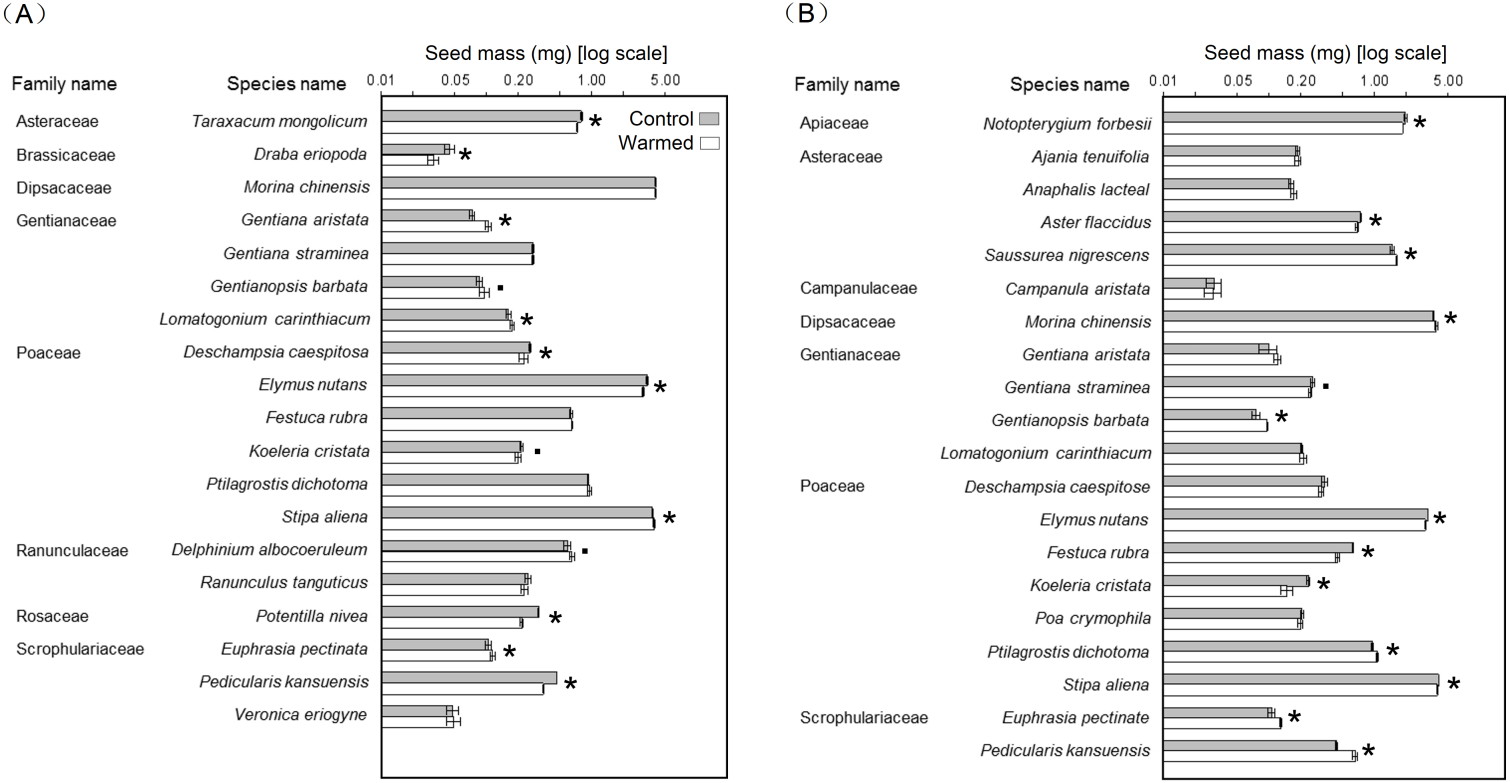
Effects of warming on mean seed mass in the LG (A) and HG (B) meadow sites. The error bars indicate ± SE; *n* = 5. The asterisks (*) and points (.) indicate the significant differences (*P* < 0.05) and marginally significant differences (*P* < 0.1) between control and warming treatments, respectively. The significance was tested using the Wilcoxon rank sum test.

We did not find a significant effect of warming on mean seed mass across all species in both sites (Table 1), however, the significant treatment × species interaction indicates that the effect is species-specific. We found that warming significantly increased the mean seed mass of *Euphrasia pectinata*, *Gentiana aristata*, *Lomatogonium carinthiacum*, and *Stipa aliena* (4 of 19 species), while decreasing the mean seed mass of *Deschampsia caespitosa*, *Draba eriopoda*, *Elymus nutans*, *Pedicularis kansuensis*, *Potentilla nivea*, and *Taraxacum mongolicum* (6 of 19 species) in the LG site (Fig. 2A).

**Table 1:** Results of the linear mixed-effect model which was used to assess the effect of warming on seed mass across all species in both LG and HG sites.

	*Df*	*F-value*	*P*
treatment	1	1.14	0.2865
species	25	2409.33	<0.0001
treatment × species	25	7.17	<0.0001

Warming significantly increased the mean seed mass of *E. pectinata*, *Gentianopsis barbata*, *Morina chinensis*, *P. kansuensis*, *Ptilagrostis dichotoma*, and *Saussurea nigrescens* (6 of 20 species), while decreasing the mean seed mass of *Aster flaccidus*, *E. nutans*, *Festuca rubra*, *Koeleria cristata*, *Notopterygium forbesii*, and *S. aliena* (6 of 20 species) in the HG site (Fig. 2B).

There were significant site × warming interactions for *E. nutans*, *F. rubra*, *K. cristata*, *M. chinensis*, *P. kansuensis*, *P. dichotoma*, and *S. aliena*, respectively (Table 2).

**Table 2:** Results of two-way ANOVA including site and warming treatment for each of the species that were found in both LG and HG sites.

Species	Warm	Site	Warm × Site
	*F*_1,16_	*F*_1,16_	*F*_1,16_
*Deschampsia caespitosa*	14.20^**^	97.46^***^	0.27
*Elymus nutans*	101.42^***^	12.67^**^	9.37^**^
*Euphrasia pectinata*	29.86^***^	5.83^*^	4.02
*Festuca rubra*	150.20^***^	218.50^***^	207.40^***^
*Gentiana aristata*	32.32^***^	19.34^***^	2.14
*Gentiana straminea*	3.36	48.09^***^	2.37
*Gentianopsis barbata*	25.57^***^	3.54	3.93
*Koeleria cristata*	57.65^***^	9.04^**^	32.59^***^
*Lomatogonium carinthiacum*	7.04^*^	80.23^***^	1.12
*Morina chinensis*	9.53^**^	92.25^***^	13.86^**^
*Pedicularis kansuensis*	16.72^***^	404.78^***^	62.91^***^
*Ptilagrostis dichotoma*	23.07^***^	21.18^***^	9.25^**^
*Stipa aliena*	0.30	85.47^***^	35.15^***^

**Notes.**

* *P* < 0.05.

** *P* < 0.01.

*** *P* < 0.001.

## Discussion

In this study, we found that long-term warming can shift the mean seed mass at a species level. However, the direction of this variation is species-specific. We found that warming increased the mean seed mass of 4 of 19 species in the LG meadow and 6 of 20 species in the HG meadow, which support hypothesis I. Moreover, we also found that warming decreased the mean seed mass of 6 of 19 species and 6 of 20 species in the LG and HG meadows, respectively, which support hypothesis II. Thus, the potential for greater energy input and life-history theory in combination determined variation in the mean seed mass of species facing warming in an alpine environment, where plants growth and seeds production are strongly limited by low temperatures (Körner, 2003; Wang et al., 2008). In other words, the two above mechanisms behind hypothesis I and II may act together and their relative contribution determines changes of mean seed mass under warming. Specifically, under the background of global warming, our study suggests that mean seed mass of alpine plant species appears to decrease in warmer (less stressful) habitats based on life-history theory, but it also suggests there may be an underlying trade-off in which mean seed mass may increase due to the potential for greater energy input. Even though species may inhabit the same community, they do not necessarily face the same physical and biotic environment (Wang et al., 2008). These differences in physical and biotic environments changing relative contribution of the two above mechanisms may result in complex patterns of variation in mean seed mass of alpine plant species facing global warming.

We also reported the interesting result that, for some species, the effect of warming on mean seed mass differed between sites. Particularly, the direction of mean seed mass variation of two species is opposite in LG and HG meadow communities. Mean seed mass of *Pedicularis kansuensis* decreased due to warming in the LG meadow, but increased in the HG meadow. Mean seed mass of *Stipa aliena* increased due to warming in the LG meadow, but decreased in the HG meadow. These patterns may be due to different environmental factors (e.g., soil chemical properties) and biotic interactions in these sites. We found that rapidly available nitrogen and rapidly available phosphorus at shallow soil depths (0–10 cm) were both higher in the LG meadow than the HG meadow, while rapidly available phosphorus at 10–20 cm was lower in the LG meadow than the HG meadow (Table S4). Our previous study found significant changes in species composition between LG and HG meadows (Zhang et al., 2017). For example, compared with the LG meadow, forbs (e.g., *Ajania tenuifolia*, *Aster flaccidus* and *Saussurea nigrescens*) were more abundant in the HG meadow. For those species having a decreased seed mass with warming in the HG meadow, it is possible that high grazing history may have released such species from some form of competition or selective pressure, such that it became favorable for them to produce smaller seeds in greater numbers (hypothesis II). In contrast, for species which had increased seed mass with warming under the HG meadow, it is possible that high grazing history increased stress for those individuals, making it favorable to invest extra energy from warming into larger, more stress-tolerant seeds (hypothesis I). In sum, differences in the physical and biotic environment in two sites may regulate the trade-off between the seedling’s chance of survival and mean seed mass, and furthermore induce different variation in mean seed mass of alpine plant species facing environmental warming.

Previous studies have demonstrated correlated evolution among seed/fruit size and other plant trait (e.g., plant height) at a species level (Herrera, 2002; Wright et al., 2007). Other functional traits (e.g., height) may influence the response of mean seed mass to warming. For example, plant height is a major determinant of a species’ ability to compete for light (Moles et al., 2009). Previous study has shown that warming tends to increase plant height, probably resulting in more intense competition for light under warming (Hudson, Henry & Cornwell, 2011). As a result, seed of low plants may become larger at warmer condition to compensate for weak light competitiveness. In addition, closely related species tend to share similar traits (e.g., seed size) or niche preferences (Losos, 2008). Moreover, closely related species may have similar responses of seed size to experimental warming. Therefore, we conclude that future studies should consider the effects of functional traits and phylogeny on variation in mean seed mass with the background of global warming.

One caveat of our study is that the sites are not replicated within grazing history treatments. This is the first simulated warming experiment on the Qinghai-Tibet Plateau. In 1990s, funding and harsh environmental conditions limited the scale of the experiment on the Tibetan Plateau. Despite this weakness, this experiment is very valuable for studying the impact of global change on the Qinghai-Tibet Plateau, and many important results (e.g., Klein, Harte & Zhao, 2004; Klein, Harte & Zhao, 2005; Wang et al., 2014a; Zhang et al., 2017) were reported based on this experiment. Our research is also of great significance for studying the influence of long-term experimental warming on plant functional traits.

## Conclusions

We found species-specific changes in mean seed mass but no overall mean seed mass changes in response to long-term experimental warming. The physical and biotic environment may modify the responses of mean seed mass to long-term experimental warming. Previous studies found that intra-specific correlations between seed mass and elevation vary between species (Wang et al., 2014b; Olejniczak et al., 2018). Olejniczak et al. (2018) suggested that we should not expect a single universal pattern in the effects of external factors on plant characteristics. Our findings also conveys this important general ecological message. Furthermore, our study suggests that under the global climate change, multiple mechanisms on variation in plant traits (e.g., seed mass) within different species may coexist, and that should be considered when interpreting community structure and function from functional traits. In this study, we gave some general explanations for variation in mean seed mass under long-term experimental warming based on life-history theory and other hypotheses. However, life history, functional traits, phylogeny, interactions with other species and soil requirements may influence the response of mean seed mass to warming. In the future, there is a need to consider the combined effects of these factors on variation in mean seed mass with the background of global warming.

##  Supplemental Information

10.7717/peerj.7416/supp-1 Data S1 Raw data

## Additional Information and Declarations

### Data Availability

The following information was supplied regarding data availability: The raw measurements are available in the Supplemental Files. The raw data shows species with their seed mass. Zhang, Chunhui; Ma, Zhen; Zhou, Huakun; Zhao, Xinquan (2019): Long-term warming results in species-specific shifts in seed mass in alpine communities. figshare. Dataset. https://doi.org/10.6084/m9.figshare.8851787.v2.
